# Conservative management of petrous apex abscess and Gradenigo's syndrome in a diabetic patient: Case report and literature review

**DOI:** 10.1002/ccr3.3625

**Published:** 2020-12-08

**Authors:** Guilherme Correa Guimaraes, Paola Piva de Freitas, Vagner Antonio Rodrigues da Silva, Arthur Menino Castilho

**Affiliations:** ^1^ Department of Otorhinolaryngology State University of Campinas Campinas Brazil

**Keywords:** case report, diabetes, malignant otitis externa, necrotizing otitis externa, petrous abscess, petrous apicitis

## Abstract

The case reported evidences the possibility of nonsurgical treatment in patients with abscesses located in regions difficult to reach surgically due to surgical limitations or possible sequelae related to surgical procedure.

## Graphical abstract

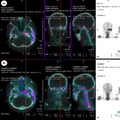

## 1 INTRODUCTION

Malignant otitis externa (MOE) is an uncommon entity with high morbidity and mortality defined as a progressive infection of the external auditory canal causing an osteomyelitis of the temporal bone.^1^, ^2^ The spreading patterns of skull base osteomyelitis can be differentiated in four compartments with the associated soft tissues and bone structures.^3^ The anterior pattern comprises the retrocondylar fat, masticator space and muscles, parotid gland, facial nerve, temporal fossa, temporomandibular joint, and styloid foramen.^3^ Posterior pattern is associated with the mastoid process.^3^ Medial or crossed pattern encompasses parapharyngeal fat, nasopharyngeal muscles and wall, glossopharyngeal nerve, vagal nerve, accessory nerve, sphenoid clivus, petrous apex, and jugular foramen.^3^ Intracranial pattern includes sigmoid sinus, jugular vein, internal carotid artery, dura mater, jugular fossa, and petroclival synchondrosis.^3^

Typical presentation includes severe otalgia and otorrhoea, and cranial nerve palsy can concomitant occur as well as conductive hearing loss.^1^, ^4^ *Pseudomonas aeruginosa* is the most frequent pathogen described, followed by *Staphylococcus aureus*.^1^, ^2^, ^5^ *Diabetes mellitus patients*, immunocompromised patients, and elderly are known to be the most endangered groups.^6^, ^7^, ^8^ Recent reports showed a strong association between *diabetes mellitus* and MOE,^1^, ^2^ although *diabetes mellitus* is a well‐established risk factor, the role of the glycemic control still remains unclear.^8^, ^9^, ^10^ Diagnosis is made correlating the clinical features with findings in high‐resolution computed tomography (HR‐TC) scans, magnetic resonance imaging (MRI), and technetium scintigraphy, often showing local tissue swelling and extensive diffuse bone destruction.^3^, ^6^, ^9^, ^11^ The follow‐up is habitually made with HR‐CT scans, gallium scintigraphy, and positron emission tomography‐computed tomography (PET‐CT) usually every 6 weeks until no evidence of remaining disease is found.^3^, ^6^, ^11^ Inflammatory markers such as erythrocyte sedimentation rate and C‐reactive protein are also useful to follow response to treatment.^6^

Gradenigo's syndrome is defined as a triad including severe retro‐orbital pain, ipsilateral abducens nerve palsy, and purulent otorrhoea, secondary to petrous apicitis.^11^, ^12^ In addition to the three classic symptoms presented in the triad, the patients with Gradenigo's syndrome may also show otalgia, fever, coma, and palsy of other cranial nerves, such as V, VII, VIII, and X.^13^ Pathophysiology of Gradenigo's syndrome lies on the inflammation of Dorello's canal and Meckel Cave, causing edema of abducens and trigeminal nerves, culminating in neuropraxia.^13^

The actual clinical treatment protocol for MEO and Gradenigo's syndrome is based on the association of a quinolone with a third‐generation cephalosporin, frequently ciprofloxacin with ceftazidime.^3^, ^4^, ^6^, ^9^ Treatment length should be no <6 weeks, which is the period of time the temporal bone needs to revascularize itself.^14^ The use of oral corticosteroids with strict glycemic control is indicated when there is severe otalgia despite appropriate treatment or when the patient presents cranial nerve palsy.^9^ Plenty of authors sought to verify the association of the involvement of cranial nerves palsy with worse prognosis; however, the correlation still remains unclear.^7^, ^9^, ^10^

## 2 CASE REPORT

A 63‐year‐old female patient presented intense left‐sided otalgia, otorrhoea, retro‐orbital pain, and diplopia initiated suddenly a week ago. No other neurological symptoms were referred. The patient denied similar previous symptoms and history of tubercular disease, reporting previous medical history of arterial hypertension and non–insulin‐dependent *diabetes mellitus* with adequate glycemic control.

At admission, the patient was afebrile, showing gaze palsy with specific limitation of left eye's abduction movement. Vital signs were normal. The otoscopy revealed at the left side purulent otorrhea with hyperemic and swollen external ear canal, in addition to a posterior lesion in the external conduct suggestive of granuloma and intact tympanic membrane; right ear was normal. No clinical signs of acute mastoiditis were found. Clinical evaluation of the cranial nerves confirmed palsy of the left eye in outward gaze, suggesting dysfunction of the left abducens cranial nerve. The III, IV, VII, IX, X, and XI cranial nerve function was bilaterally preserved. An otorrhea sample was sent to culture.

Blood analysis revealed WBC 9.190/mm^3^, CRP 74.60 mg/L, and glycated hemoglobin 6.4%. HIV test was negative. Audiometric evaluation showed left mild conductive hearing loss with pure tone average (PTA, calculated at 0.5, 1, 2, 4 kHz) of 13.75 dB HL in the right ear and 21.25 dB HL in the left side. Speech audiometry confirmed left hearing loss.

High‐resolution computed tomography (HR‐CT) was performed, showing no evidence of stroke and an opacification of the left mastoid cells. ENT counseling was requested.

Lumbar puncture, performed on the third day of admission, ruled out infectious etiologies. CRP levels were 5.12 mg/L at the end of the first week of treatment. Culture result was available, on the 9th day of admission revealing *Proteus mirabilis*, *Alcaligenis faecalis*, *Enterococcus faecalis*, *Citrobacter koseri,* and *Bordetella trematum*.

**FIGURE 1:**
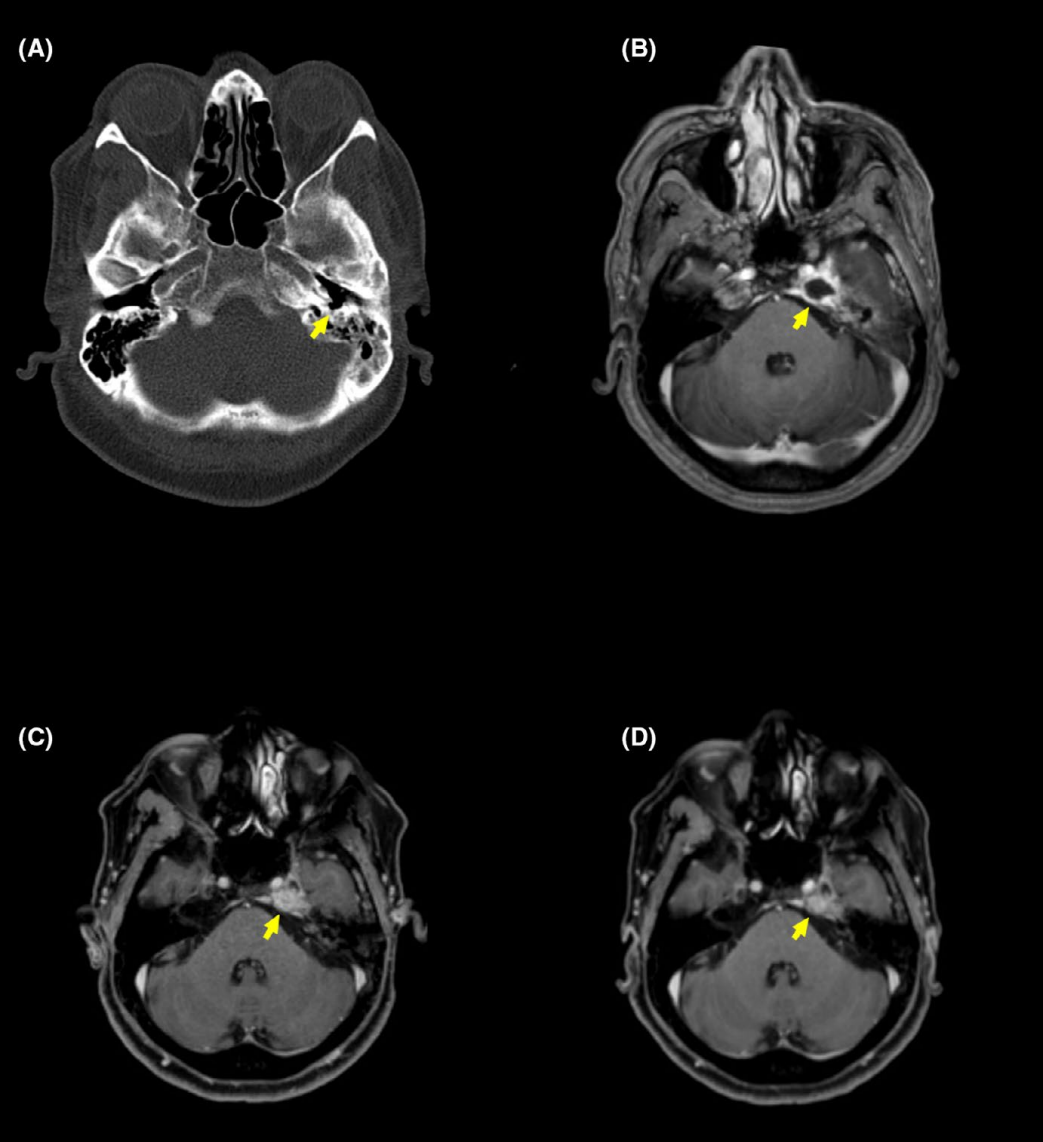
Imaging scans performed during patient's evaluation. A, Brain HR‐CT scan showing a bone gap (arrow) in the posterior segment of the external auditory canal. B, Brain MRI performed on day 8 pointing an inflammatory process in the left mastoid associated with an 2 cm abscess (arrow) in the left petrous apex extending to the dura mater. C, Brain MRI performed on the 33rd day pointing an improvement of the inflammatory process in the left mastoid‐associated abscess size reduction (arrow). D, Brain MRI performed 52 d after the first brain HR‐CT scan. Brain MRI was performed 8 d after discharge to evaluate response to treatment, showing no evidence of central sinus thrombosis, complete abscess resolution (arrow), absence of the signs of insinuation to the left pontocerebellar cistern, and partial obliteration of Meckel's cave

**FIGURE 2:**
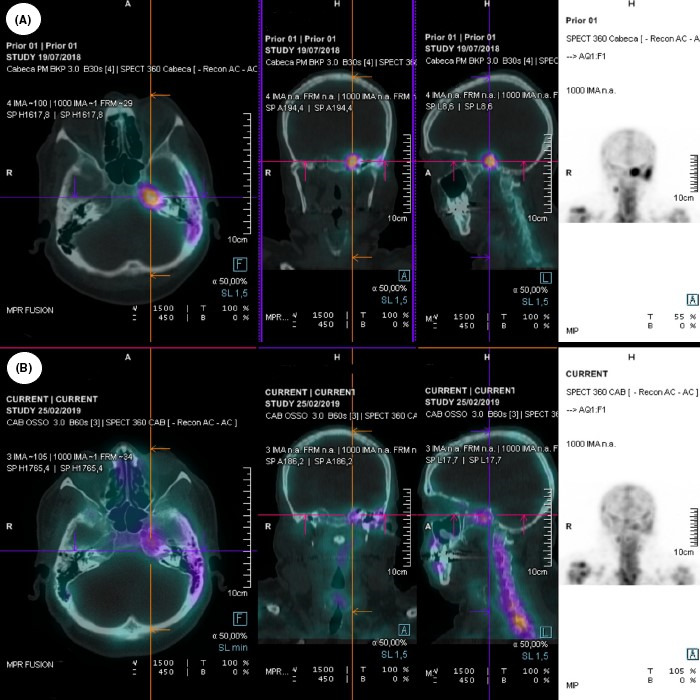
Technetium scintigraphy. A, First technetium scintigraphy showing hypercapture in the left mastoid and petrous apex suggesting inflammatory process. B, Interval technetium scintigraphy at the follow‐up showing resolution of the inflammatory process in the left temporal bone

Technetium scintigraphy was performed 2 months after hospitalization, showing a bone gap in the posterior wall of external auditory canal (Figure 1A) and hypercapture in the left temporal bone with extension to the sphenoid bone ipsilaterally (Figure 2A). A brain magnetic resonance imaging (brain MRI) was performed on the 8th day of admission (Figure 1B), revealing T1 isointensity of the left mastoid with partial substitution of the normal hyperintensity of the clival bone marrow due to inflammation. The left petrous apex was hyperintense in T2‐weighted scans. After contrast administration, an enhancing rim delineated the presence of a 2 cm diameter abscess in the petrous apex extending to the dura mater and insinuating to the left pontocerebellar cistern, causing anteriorly the obliteration of Meckel's cave and involving the cavernous sinus with no signs of thrombosis. At this point, blood examinations revealed WBC 5.060/mm^3^ and CRP 6.55 mg/L. Neurosurgical team opted for a nonsurgical treatment due to surgery risks, the difficulty to access the abscess location, and the good outcomes the patient presented at the moment.

On the 33rd day of admission, an interval brain MRI was performed (Figure 1C) showing inflammatory process improvement in the petrous apex with signs of cavernous sinus thrombosis, so the patient was anticoagulated with enoxaparin. White blood cell count was 4.940/mm^3^, and CRP was 2.12 mg/L. On the 37th day, a new audiogram was executed showing a partial improvement of the left mild conductive hearing loss with PTA of 13.75 dB HL in the right ear and 18.75 dB HL in the left side.

Interval technetium scintigraphy was performed prior to discharge (Figure 2B), on day 53, showing hypercapture in the left mastoid and petrous apex suggesting inflammatory process. At the discharge, blood examinations revealed WBC 4.740/mm^3^ and CRP 1.62 mg/L. Interval brain MRI was performed 8 days after discharge to evaluate response to treatment (Figure 1D), showing no evidence central sinus thrombosis, complete abscess resolution, no signs of insinuation to the left pontocerebellar cistern, and maintaining a partial obliteration of Meckel's cave.

A weekly outpatient follow‐up was made, and antibiotic therapy was suspended 1 month after discharge. Interval technetium scintigraphy to evaluate response to treatment was performed at the end of the treatment showing no evidence of active inflammatory process in the left temporal bone (Figure 2B). The patient started to show ocular motricity enhancement and diplopia improvement after discharge, with total left abducens nerve palsy recovery 4 months after the first symptom presentation.

### 2.1 Treatment course

The patient was hospitalized, and intravenous antibiotic therapy with combined scheme of ceftriaxone and ciprofloxacin was started with ceftriaxone 1 g twice a day associated with ciprofloxacin 500 mg twice a day. Ceftriaxone was changed to ceftazidime 1 g every 8 hours due to the lack of neurological infection evidence, as the local protocol to treat MEO recommends, in addition to topical antibiotic ear drops twice a day (combination of gentamicin and betamethasone).^4^, ^6^, ^15^ No corticosteroid was administered due to difficult in glycemic control.

The antibiotic regimen was readjusted on the 9th day due to evidence of abscess in the brain MRI and swab results to ampicillin 2G every 4 hours, metronidazole 500 mg every 8 hours, and ceftazidime 2 g every 8 hours. On the 27th day of admission, the topical antibiotic was changed to a combination of ciprofloxacin and hydrocortisone twice a day.

The patient was discharged after 54 days of hospitalization with a prescription of ciprofloxacin 500 mg twice a day associated with a topical ear drop combination of ciprofloxacin and hydrocortisone.

## 3 DISCUSSION

Petrous apicitis is a rare, but life‐threatening, entity associated with acute and chronic inflammatory process in the temporal bone. The pathophysiology relying on two different possibilities based on the pneumatization grade of the temporal bone is as follows: by contiguous evolution secondary to and middle ear infection in pneumatized temporal bones or by hematogenous spread in less pneumatized temporal bones.^16^

The treatment for petrous apicitis still remains controversial between exclusively clinical treatment and treatment associated with surgery. Surgical interventions as an adjuvant treatment modality are not a consensus. Some authors advocate as an option for patients with the absence of improvement with clinical treatment or when bad prognosis factors are present, such as abscess formation or osteonecrosis.^6^, ^7^, ^13^, ^17^

Petrous apex abscess surgical treatment is a controversial topic. As Savasta et al^16^ presented, from all pediatric patients with petrous abscess, only about 25% was submitted to direct drainage, being more common than other surgical interventions such as simple mastoidectomy or ventilation tube insertion. Surgical modalities are chosen based on preoperative hearing status, temporal bone anatomy, and surgeon’s experience.^13^ The surgical risks change between the different approaches, but in general include hearing loss, facial nerve palsy, intracranial thrombosis, and intracranial infection.^18^ As Gadre and Chole said, the shortest least morbid route should be chosen, preserving when possible the hearing and the facial nerve.^11^ Surgical approach also gives the possibility of looking for granulomatous disease, obtain culture, and search for tumors.^11^

## 4 CONCLUSION

There is no management consensus, varying from petrosectomy to nonsurgical medical treatment. The case reported evidences a successful nonsurgical treatment in a patient with a 2 cm petrous apex abscess associated with left abducens nerve palsy, secondary to MEO. The patient also presented the full recovery of all deficits and abscess resolution. Nonsurgical treatment was opted based on the difficulty to access the abscess location in a surgical procedure and surgical sequelae, but the author highlights that every patient should receive an individualized evaluation and personal therapeutic proposal.

## CONFLICTS OF INTEREST

The authors declare no conflicts of interest.

## AUTHOR CONTRIBUTIONS

GCG: served as patient's physician, reviewed the literature, and contributed to manuscript drafting. PPdF: served as patient's physician, reviewed the literature, and contributed to manuscript drafting. VARdS: responsible for the revision of the manuscript and for important intellectual content during the manuscript drafting. AMC: responsible for the revision of the manuscript and for important intellectual content during the manuscript drafting.

## Funding information

State University of Campinas—UNICAMP.

## ETHICAL APPROVAL

Patients' consent was obtained.

## Data Availability

The data that support the findings of this study are available from the corresponding author upon request.
